# Quinazoline-2,4(1*H*,3*H*)-dione

**DOI:** 10.1107/S1600536808024240

**Published:** 2008-08-06

**Authors:** Ge Liu

**Affiliations:** a Chifeng University, Chifeng 024000, People’s Republic of China

## Abstract

In the title compound, C_8_H_6_N_2_O_2_, inter­molecular N—H⋯O hydrogen bonds involving the amine and carbonyl groups create centrosymmetric dimers between adjacent nearly coplanar mol­ecules. These dimers are further connected by weak N—H⋯O hydrogen bonds, forming a two-dimensional network. Mol­ecules are packed in the crystal structure with adjacent benzene and pyrimidine rings approximately coplanar; the centroid–centroid separation is 3.863 Å and the dihedral angle between the mean planes is 0.64°, indicating the presence of weak inter­molecular face-to-face π–π stacking inter­actions.

## Related literature

For background, see: Goto *et al.* (1993 ▶); Mohri (2001 ▶); For further synthetic details, see: Mizuno *et al.* (2007 ▶).
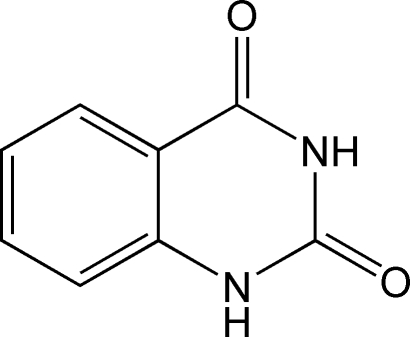


## Experimental

### 

#### Crystal data

C_8_H_6_N_2_O_2_
*M* *_r_* = 162.15
Monoclinic, 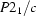

*a* = 10.891 (2) Å
*b* = 5.2810 (11) Å
*c* = 12.701 (3) Å
β = 99.61 (3)°
*V* = 720.2 (3) Å^3^
*Z* = 4
Mo *K*α radiation
μ = 0.11 mm^−1^
*T* = 293 (1) K
0.20 × 0.18 × 0.15 mm

#### Data collection

Rigaku R-AXIS RAPID-S diffractometer
Absorption correction: none
5683 measured reflections
1262 independent reflections
869 reflections with *I* > 2σ(*I*)
*R*_int_ = 0.051

#### Refinement

*R*[*F* ^2^ > 2σ(*F* ^2^)] = 0.055
*wR*(*F* ^2^) = 0.103
*S* = 1.06
1262 reflections
110 parameters
H-atom parameters constrained
Δρ_max_ = 0.12 e Å^−3^
Δρ_min_ = −0.12 e Å^−3^

### 

Data collection: *RAPID-AUTO* (Rigaku, 1998 ▶); cell refinement: *RAPID-AUTO*; data reduction: *CrystalStructure* (Rigaku/MSC, 2002 ▶); program(s) used to solve structure: *SHELXS97* (Sheldrick, 2008 ▶); program(s) used to refine structure: *SHELXL97* (Sheldrick, 2008 ▶); molecular graphics: *SHELXTL* (Sheldrick, 2008 ▶); software used to prepare material for publication: *SHELXTL*.

## Supplementary Material

Crystal structure: contains datablocks I, global. DOI: 10.1107/S1600536808024240/bh2183sup1.cif

Structure factors: contains datablocks I. DOI: 10.1107/S1600536808024240/bh2183Isup2.hkl

Additional supplementary materials:  crystallographic information; 3D view; checkCIF report

## Figures and Tables

**Table 1:** Hydrogen-bond geometry (Å, °)

*D*—H⋯*A*	*D*—H	H⋯*A*	*D*⋯*A*	*D*—H⋯*A*
N2—H2*B*⋯O1^i^	0.86	2.00	2.854	176
N1—H1*A*⋯O1^ii^	0.86	2.13	2.976	168

Symmetry codes: (i) 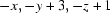; (ii) 
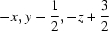
.

**Fig. 1.**
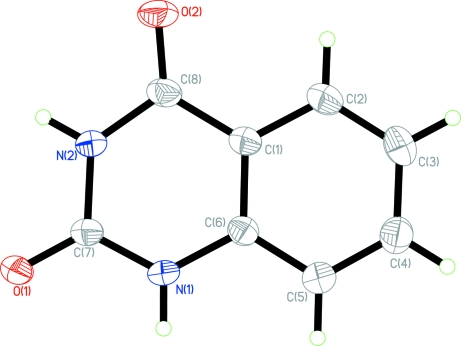
The structure of the title compound with 30% displacement probability ellipsoids.

**Fig. 2.**
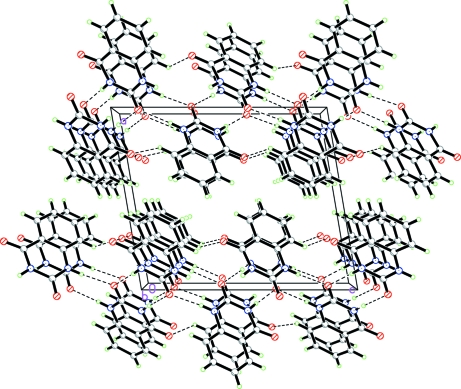
Two dimensional sheet formed by hydrogen bonds (dashed lines) in the title compound.

## supplementary crystallographic information

### Comment

2,4(1*H*,3*H*)-Quinazolinedione derivatives are of interest because of their biological activity, and they have been widely used as key structures in medicinal drugs (Goto *et al.*, 1993; Mohri, 2001; Mizuno *et al.*, 2007). We herein report the crystal structure of the title compound (I). In the molecule (Fig. 1), the bond lengths and angles are within normal ranges. Intermolecular N—H···O hydrogen bonds involving amine NH and carbonyl groups O atoms form a two dimensional network (Table 1 and Fig. 2). Weak π–π stacking interactions are also observed in the crystal structure.

### Experimental

To a 100-ml, 3-necked flask equipped with condenser, were added 2-aminobenzonitrile (5.91 g, 50 mmol) and 1,8-diazabicyclo(5.4.0)undec-7-ene (DBU, 1.50 ml, 10 mmol) under argon, together with a large magnetic stirring bar. Then, CO_2_ (1 bar) was charged at 293 K. The mixture was vigorously stirred under CO_2_ (1 bar) at 423 K for 4 h. The resulting white solid was then poured into 1 *M* HCl (100 ml) and washed with *t*-BuOMe (200 ml) to give pure (I). Single crystals of (I) were obtained from a water solution, at room temperature, by slow evaporation.

### Refinement

All H atoms were placed in geometrically idealized positions and constrained to ride on their parent atoms, with N—H = 0.86 Å, C—H = 0.93 Å, and with *U*_iso_(H) = 1.2*U*_eq_(parent atom).

### Crystal data

**Table:**

C_8_H_6_N_2_O_2_	*F*_000_ = 336
*M**_r_* = 162.15	*D*_x_ = 1.495 Mg m^−^^3^
Monoclinic, *P*2_1_/*c*	Mo *K*α radiation λ = 0.71073 Å
Hall symbol: -P 2ybc	Cell parameters from 5473 reflections
*a* = 10.891 (2) Å	θ = 3.3–27.5º
*b* = 5.2810 (11) Å	µ = 0.11 mm^−^^1^
*c* = 12.701 (3) Å	*T* = 293 (1) K
β = 99.61 (3)º	Block, colorless
*V* = 720.2 (3) Å^3^	0.20 × 0.18 × 0.15 mm
*Z* = 4	

### Data collection

**Table:**

Rigaku R-AXIS RAPID-S diffractometer	869 reflections with *I* > 2σ(*I*)
Radiation source: fine-focus sealed tube	*R*_int_ = 0.051
Monochromator: graphite	θ_max_ = 25.0º
*T* = 293(1) K	θ_min_ = 3.3º
ω scans	*h* = −12→12
Absorption correction: none	*k* = −6→6
5683 measured reflections	*l* = −15→15
1262 independent reflections	

### Refinement

**Table:**

Refinement on *F*^2^	Hydrogen site location: inferred from neighbouring sites
Least-squares matrix: full	H-atom parameters constrained
*R*[*F*^2^ > 2σ(*F*^2^)] = 0.055	*w* = 1/[σ^2^(*F*_o_^2^) + (0.0327*P*)^2^ + 0.2501*P*] where *P* = (*F*_o_^2^ + 2*F*_c_^2^)/3
*wR*(*F*^2^) = 0.103	(Δ/σ)_max_ = 0.004
*S* = 1.07	Δρ_max_ = 0.12 e Å^−^^3^
1262 reflections	Δρ_min_ = −0.12 e Å^−^^3^
110 parameters	Extinction correction: SHELXL97 (Sheldrick, 2008), Fc^*^=kFc[1+0.001xFc^2^λ^3^/sin(2θ)]^-1/4^
Primary atom site location: structure-invariant direct methods	Extinction coefficient: 0.009 (3)
Secondary atom site location: difference Fourier map	

### Fractional atomic coordinates and isotropic or equivalent isotropic displacement parameters (Å^2^)

**Table:**

	*x*	*y*	*z*	*U*_iso_*/*U*_eq_	
C1	0.2762 (2)	0.9421 (4)	0.57398 (17)	0.0395 (6)	
N1	0.11953 (17)	1.0471 (4)	0.67998 (14)	0.0453 (6)	
H1A	0.0866	1.0176	0.7357	0.054*	
O1	−0.01889 (16)	1.3655 (3)	0.62957 (12)	0.0546 (5)	
C2	0.3772 (2)	0.7919 (5)	0.5574 (2)	0.0494 (7)	
H2A	0.4148	0.8190	0.4977	0.059*	
N2	0.12516 (17)	1.2702 (4)	0.52514 (14)	0.0442 (6)	
H2B	0.0924	1.3842	0.4809	0.053*	
O2	0.26963 (17)	1.2019 (3)	0.41910 (13)	0.0633 (6)	
C3	0.4210 (2)	0.6050 (5)	0.6289 (2)	0.0560 (8)	
H3A	0.4884	0.5060	0.6179	0.067*	
C4	0.3646 (2)	0.5637 (5)	0.7176 (2)	0.0540 (7)	
H4A	0.3943	0.4359	0.7655	0.065*	
C5	0.2655 (2)	0.7091 (5)	0.7357 (2)	0.0502 (7)	
H5A	0.2287	0.6809	0.7956	0.060*	
C6	0.2210 (2)	0.8984 (4)	0.66362 (17)	0.0388 (6)	
C7	0.0702 (2)	1.2336 (5)	0.61374 (18)	0.0419 (6)	
C8	0.2277 (2)	1.1435 (5)	0.49911 (18)	0.0444 (6)	

### Atomic displacement parameters (Å^2^)

**Table:**

	*U*^11^	*U*^22^	*U*^33^	*U*^12^	*U*^13^	*U*^23^
C1	0.0414 (14)	0.0406 (14)	0.0384 (13)	−0.0030 (12)	0.0121 (11)	−0.0038 (12)
N1	0.0517 (13)	0.0533 (13)	0.0344 (11)	0.0086 (11)	0.0179 (10)	0.0109 (10)
O1	0.0556 (11)	0.0681 (13)	0.0450 (10)	0.0219 (10)	0.0221 (8)	0.0109 (9)
C2	0.0500 (16)	0.0520 (17)	0.0499 (16)	0.0001 (14)	0.0191 (13)	−0.0075 (14)
N2	0.0538 (13)	0.0474 (13)	0.0346 (11)	0.0079 (10)	0.0169 (10)	0.0077 (10)
O2	0.0853 (14)	0.0669 (13)	0.0469 (10)	0.0062 (11)	0.0380 (10)	0.0071 (10)
C3	0.0486 (16)	0.0527 (17)	0.0670 (19)	0.0098 (14)	0.0107 (15)	−0.0099 (16)
C4	0.0556 (17)	0.0519 (17)	0.0532 (16)	0.0063 (14)	0.0049 (14)	0.0017 (14)
C5	0.0523 (16)	0.0550 (17)	0.0445 (15)	0.0044 (14)	0.0117 (12)	0.0057 (13)
C6	0.0385 (14)	0.0404 (14)	0.0378 (13)	−0.0006 (12)	0.0077 (11)	−0.0037 (12)
C7	0.0456 (15)	0.0482 (15)	0.0336 (13)	0.0018 (13)	0.0118 (12)	0.0019 (12)
C8	0.0547 (17)	0.0442 (15)	0.0379 (14)	−0.0022 (13)	0.0181 (12)	−0.0070 (12)

### Geometric parameters (Å, °)

**Table:**

C1—C6	1.393 (3)	N2—C8	1.389 (3)
C1—C2	1.401 (3)	N2—H2B	0.8600
C1—C8	1.465 (3)	O2—C8	1.221 (3)
N1—C7	1.348 (3)	C3—C4	1.388 (3)
N1—C6	1.399 (3)	C3—H3A	0.9300
N1—H1A	0.8600	C4—C5	1.375 (3)
O1—C7	1.238 (3)	C4—H4A	0.9300
C2—C3	1.372 (3)	C5—C6	1.387 (3)
C2—H2A	0.9300	C5—H5A	0.9300
N2—C7	1.373 (3)		
			
C6—C1—C2	119.1 (2)	C5—C4—C3	120.9 (3)
C6—C1—C8	119.6 (2)	C5—C4—H4A	119.5
C2—C1—C8	121.3 (2)	C3—C4—H4A	119.5
C7—N1—C6	124.0 (2)	C4—C5—C6	119.3 (2)
C7—N1—H1A	118.0	C4—C5—H5A	120.3
C6—N1—H1A	118.0	C6—C5—H5A	120.3
C3—C2—C1	120.2 (2)	C5—C6—C1	120.5 (2)
C3—C2—H2A	119.9	C5—C6—N1	120.3 (2)
C1—C2—H2A	119.9	C1—C6—N1	119.2 (2)
C7—N2—C8	127.2 (2)	O1—C7—N1	123.4 (2)
C7—N2—H2B	116.4	O1—C7—N2	121.0 (2)
C8—N2—H2B	116.4	N1—C7—N2	115.6 (2)
C2—C3—C4	119.9 (2)	O2—C8—N2	120.2 (2)
C2—C3—H3A	120.0	O2—C8—C1	125.4 (2)
C4—C3—H3A	120.0	N2—C8—C1	114.3 (2)
			
C6—C1—C2—C3	−0.1 (4)	C7—N1—C6—C1	0.0 (3)
C8—C1—C2—C3	179.8 (2)	C6—N1—C7—O1	−179.3 (2)
C1—C2—C3—C4	0.3 (4)	C6—N1—C7—N2	0.9 (3)
C2—C3—C4—C5	−0.5 (4)	C8—N2—C7—O1	177.6 (2)
C3—C4—C5—C6	0.4 (4)	C8—N2—C7—N1	−2.7 (3)
C4—C5—C6—C1	−0.3 (4)	C7—N2—C8—O2	−177.4 (2)
C4—C5—C6—N1	179.4 (2)	C7—N2—C8—C1	3.1 (3)
C2—C1—C6—C5	0.1 (3)	C6—C1—C8—O2	178.7 (2)
C8—C1—C6—C5	−179.9 (2)	C2—C1—C8—O2	−1.3 (4)
C2—C1—C6—N1	−179.6 (2)	C6—C1—C8—N2	−1.8 (3)
C8—C1—C6—N1	0.4 (3)	C2—C1—C8—N2	178.2 (2)
C7—N1—C6—C5	−179.7 (2)		

### Hydrogen-bond geometry (Å, °)

**Table:**

*D*—H···*A*	*D*—H	H···*A*	*D*···*A*	*D*—H···*A*
N2—H2B···O1^i^	0.86	2.00	2.854	176
N1—H1A···O1^ii^	0.86	2.13	2.976	168

Symmetry codes: (i) −*x*, −*y*+3, −*z*+1; (ii) −*x*, *y*−1/2, −*z*+3/2.
